# Genome-Wide Analysis of *Nelumbo nucifera UXS* Family Genes: Mediating Dwarfing and Aquatic Salinity Tolerance

**DOI:** 10.3390/plants15010116

**Published:** 2026-01-01

**Authors:** Li Wang, Xingyan Zheng, Yajun Liu, Qian Mao, Yiwen Chen, Lin Zhao, Xiaomao Cheng, Longqing Chen, Huizhen Hu

**Affiliations:** 1 Yunnan Key Laboratory of Landscape Plant Resource Cultivation and Application, College of Landscape Architecture and Horticulture Sciences, Southwest Forestry University, Kunming 650224, China; wangli@swfu.edu.cn (L.W.); xingyanzheng@swfu.edu.cn (X.Z.); maoqian@swfu.edu.cn (Q.M.); chenyiwen17@swfu.edu.cn (Y.C.); xmcheng@swfu.edu.cn (X.C.); 2 Lincang Agricultural Technology Extension Station, Lincang 677000, China; lcsyls@163.com; 3 Lijiang Economic Crop Workstation, Lijiang 674100, China; zhao_lin202010@163.com

**Keywords:** *NnUXS* gene family, *NnUXS3*, plant architecture dwarfing, salt tolerance, lotus (*N. nucifera*)

## Abstract

*Nelumbo nucifera* (Lotus) is an economically important aquatic crop frequently challenged by abiotic stresses. The plant cell wall, a primary interface with the environment, undergoes dynamic remodeling to balance structural integrity with adaptation. UDP-glucuronic acid decarboxylase (UXS), a key enzyme synthesizing the nucleotide sugar precursor UDP-xylose, exists in distinct membrane-bound (e.g., Golgi) and cytosolic forms, channeling substrates into compartmentalized polysaccharide biosynthesis pathways and positioning the UXS family as a crucial regulator linking cell wall metabolism to plant adaptation. Here, we systematically characterized the *NnUXS* gene family in lotus through genome-wide identification, evolutionary synteny analysis, and functional validation. Integrated bioinformatic analysis revealed their physicochemical properties, motif patterns, and regulatory *cis*-elements, suggesting potential roles in growth and salt stress responses. Among the family, *NnUXS3* was prioritized due to its preferentially upregulated in small plant architecture (SPA) varieties, its early induction under salt stress (0.5 days, 200 mM NaCl), and its highest predicted binding affinity for UDP-GlcA (−8.9 kcal/mol). Subsequent functional validation confirmed its dual role: heterologous overexpression in tobacco reduced plant height (47.22%) and leaf area (67.61%), while transient overexpression in lotus enhanced salt tolerance and shortened the petioles. This enhanced tolerance was achieved by upregulating key genes involved in polysaccharide biosynthesis (*NnCSLC4*, *NnXTH22*, *NnCESA1*) and antioxidant defense (*NnSOD*, *NnPOD*). Our findings establish *NnUXS3* as a key mediator in balancing plant architecture and abiotic stress resilience. This work not only identifies a valuable genetic target for lotus breeding but also provides insights into the growth-stress trade-off, highlighting the importance of UXS subcellular localization in tailoring cell wall remodeling for environmental adaptation.

## 1. Introduction

The plant cell wall, a dynamic extracellular matrix critical for plant development and stress resilience, exhibits a hierarchical architecture: cellulose microfibrils form a crystalline backbone, embedded within and cross-linked by a network of hemicelluloses and pectins, which collectively balances mechanical strength with functional plasticity [1,2,3,4,5]. This architecture is not merely structural, the intercalation of hemicelluloses (e.g., xylans, xyloglucans) and pectins modulates wall extensibility, regulates cell elongation, and governs adaptive responses to environmental cues [6,7,8,9]. For instance, our recent analysis of fresh-cut flower stems (*Rose chinensis*, *Gerbera jamesonii*, *Dianthus carnation*, *Nymphaea tetragona*, *Eustoma grandiflorum*, and *N*. *nucifera*) revealed that hemicellulose/pectin side chains confined within cellulose microfibrils reduce cellulose crystallinity and polymerization degree, weakening mechanical rigidity and driving stem bending [6]. Fine-tuning hemicellulose-pectin-cellulose interactions is a critical mechanism for plant architecture remodeling, also as evidenced by the role of xyloglucan endotransglucosylase/hydrolases (XTHs) in determining architecture diversity in lotus [4] and the regulation of stem strength via lignin and cellulose biosynthesis in rice [10]. Furthermore, this dynamic network is pivotal for abiotic stress tolerance. Salt stress, in particular, disrupts osmotic and ionic homeostasis, impairing cell wall biosynthesis and remodeling [11,12,13]. Maintaining wall integrity through polysaccharide synthesis, such as via cellulose synthase-like proteins (e.g., SOS6/CSLD5), constitutes a vital defense layer that enhances salt tolerance by stabilizing wall structure and modulating reactive oxygen species (ROS) scavenging [14,15]. Consequently, the hemicellulose-pectin-cellulose interface serves as a central hub integrating structural support with adaptive signaling.

The biosynthesis of these wall polysaccharides relies on nucleotide sugar donors (NDPs) as essential metabolic precursors [1,16,17]. A key metabolic node in this pathway involves the decarboxylation of UDP-glucuronate (UDP-GlcA) to UDP-xylose (UDP-Xyl), catalyzed by UDP-glucuronate decarboxylase (UXS) [18,19]. UDP-Xyl is a direct precursor for hemicellulose (xylan/xyloglucan) biosynthesis and can be converted to UDP-arabinose for both hemicellulose and pectin metabolism, thereby directly shaping wall composition and properties [12,16,20,21,22,23]. The UXS enzyme, belonging to the NAD^+^ isomerase/dehydratase family, features conserved structural motifs for NAD(P) binding and a Ser-Lys-Tyr catalytic triad, facilitating the conversion of UDP-GlcA to UDP-Xyl [16,18,24]. Functional studies in model plants underscore its critical role: loss-of-function *uxs* mutants in *Arabidopsis* (*uxs3/5/6* triple mutant) and rice (*FRAGILE CULM 18/UXS3* mutant) exhibit reduced xylan content, brittle stems, and compromised growth, confirming that UXS-mediated NDP flux is an upstream controller of hemicellulose-dependent wall strength and plant integrity [22,23,24,25,26]. Despite this established importance in wall biology, the *UXS* gene family remains uncharacterized in perennial aquatic plants like lotus.

Lotus (*N. nucifera*) is an economically important aquatic crop exhibiting substantial phenotypic diversity, particularly in plant architecture, which ranges from large to prized small-plant architecture (SPA) cultivars optimized for ornamental use [4,27,28,29,30,31]. The cultivation of these SPA varieties, however, coincides with a potential need for enhanced resilience against prevalent abiotic stresses such as salinity [9,32,33,34]. Intriguingly, plants often employ a sophisticated growth-stress trade-off mechanism, where resource reallocation under stress can slow growth to enhance tolerance, suggesting intrinsic molecular links between architecture and stress adaptation [35,36,37]. Evidence supporting this coordinated regulation is found across diverse systems. For instance, in alfalfa, interference with *MsNAC73* expression not only increased branching but also enhanced salt tolerance by reducing ion toxicity and oxidative damage [38]. Similarly, in rice, the ABA-responsive transcription factor OsMYB2 mediates amino acid transport to concurrently positively regulate tillering, yield, and salt stress adaptation [39]. These examples underscore the existence of shared genetic modules that coregulate development and stress resilience. Given the dual role of the cell wall as both a structural determinant of architecture and a mediator of stress responses, we propose that key regulators of wall biosynthesis, such as UXS, are prime molecular candidates for orchestrating such coordinated adaptation. However, the specific function of the *UXS* gene family in mediating the intersection of plant architecture and abiotic stress tolerance in lotus remains entirely unexplored.

Therefore, we hypothesize that the *NnUXS* gene family mediates both plant architecture and salt tolerance in lotus by modulating cell wall polysaccharide biosynthesis. This study aims to: (1) systematically identify and characterize the *NnUXS* gene family; (2) dissect their roles in plant architecture via expression profiling in large- versus small-plant architecture varieties and functional validation; and (3) evaluate their contribution to salt tolerance. This work will elucidate a key molecular link between growth and adaptation in lotus, providing genetic resources for the targeted improvement of this valuable crop, and may offer insights into the conserved mechanisms of growth-stress trade-offs in other plant species.

## 2. Results

### 2.1. Identification of UXS Family Members in N. nucifera

Following a genome-wide search of the publicly available ‘Ancient China’ lotus genome, 28 initial candidate genes were identified. Subsequent screening for the conserved UXS domain confirmed five authentic *NnUXS* genes, which were systematically named *NnUXS1* to *NnUXS5* according to their chromosomal locations.

Fundamental properties, including genomic length, amino acid residue count, molecular weight (Mw), isoelectric point (PI), instability index, grand average of hydropathy (GRAVY), transmembrane domains (TMHs), and subcellular localization, were predicted (Table 1). Physicochemical analysis revealed that among the five NnUXS protein sequences, the shortest was NnUXS1 (encoding 414 amino acids), while the longest was NnUXS2 (encoding 455 amino acids). The molecular masses of NnUXS proteins ranged from 46.005 kDa (NnUXS1) to 50.197 kDa (NnUXS2). Isoelectric points varied, with the highest at 10.11 (NnUXS1) and the lowest at 9.59 (NnUXS2), indicating both were basic proteins. Instability indexes ranged from 33.24 (NnUXS3) to 50.72 (NnUXS1), and aliphatic indexes spanned 79.28 (NnUXS3) to 83.41 (NnUXS2). All NnUXS members were characterized as hydrophilic proteins (hydrophilicity scores < 0) and contained 1 (in NnUXS1/2/4) to 2 (in NnUXS3/5) TMHs. Subcellular localization predictions indicated *NnUXS1* and *NnUXS2* localized to the endoplasmic reticulum, *NnUXS3* and *NnUXS5* to the cytoplasm, *NnUXS4* to the nucleus, and *NnUXS1* and *NnUXS2* to the endoplasmic reticulum.

### 2.2. Phylogenetic Analysis of the NnUXS Family

**Figure 1:**
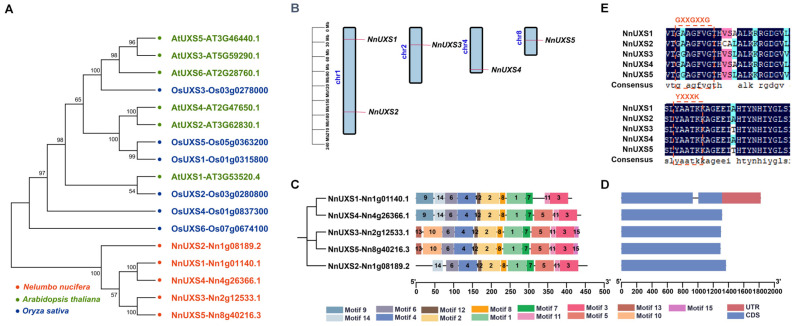
Analysis of NnUXS family members: phylogenetic tree, chromosome localization, conserved motifs and gene structure. (**A**) Phylogenetic tree of UXS proteins from *N. nucifera* (Nn), *A. thaliana* (At), and *O. sativa* (Os), using the neighbor-joining method. Red denote NnUXS, green denote AtUXS, and blue denote OsUXS. (**B**) Chromosome localization of 5 *NnUXS* genes. The scale provided represents the chromosome size (Mb). (**C**) Motif composition of *NnUXS* genes, with distinct motifs shown as colored boxes, as indicated in the scheme on the right. (**D**) Exon–intron structure of *NnUXS* genes, with UTRs and CDS indicating untranslated regions and coding sequences, respectively. (**E**) NnUXS amino acid sequence alignment. The dashed box shows the GXXGXXG and YXXXK domains of the *UXS* gene family.

To elucidate the evolutionary relationships of the *UXS* gene family, a phylogenetic tree was constructed using the full-length protein sequences of 17 members, including 6 from *Arabidopsis thaliana* (AtUXS), 6 from *Oryza sativa* (OsUXS), and 5 from *N. nucifera* (NnUXS) (Figure 1A and Tables S1–S3). The results revealed that the 17 UXS members broadly clustered into two major clades. All NnUXS genes formed a single distinct branch, whereas AtUXS and OsUXS grouped together in another branch, while OsUXS6 did not cluster with either of these two branches. This phylogenetic distribution suggests a closer evolutionary relationship between AtUXS and OsUXS, implying potentially more similar and conserved functions. Conversely, the independent clustering of NnUXS members indicates potential functional divergence and acquisition of specialized roles—possibly because lotus is an evolutionarily distinct ancient dicotyledonous plant, or due to its perennial aquatic environment differing from that of *Arabidopsis* and rice.

### 2.3. Structure Analysis of UXS Family Members in N. nucifera

Lotus genomic annotation data revealed that 5 *NnUXS* genes were primarily distributed across four chromosomes (Chr)—specifically Chr1, Chr2, Chr4, and Chr8 (Figure 1B). However, this distribution was uneven, with 2, 1, 1, and 1 genes located on these chromosomes, respectively. This unevenness may arise from non-uniform duplication events across chromosomal segments.

To characterize the structural features of NnUXS proteins, motif identification was conducted using the MEME tool. Results showed that these proteins harbored similar motif types and counts, with comparable distribution patterns. In total, 15 distinct conserved motifs were identified in the *NnUXS* proteins, each containing 11–13 motifs (Figure 1C). Specifically, NnUXS3 and NnUXS5 shared 13 common motifs, motifs 1–8, 10–13, and 15. In contrast, NnUXS1, NnUXS2, and NnUXS4 shared only 10 motifs (motifs 1–4, 6–8, 11, and 12). Notably, motifs 1–4, 6–8, and 11 were unique to all NnUXS proteins.

Furthermore, analysis of *NnUXS* gene intron–exon structures revealed the following patterns (Figure 1D). *NnUXS1* contains two exons and one intron. In contrast, *NnUXS2*-*NnUXS5* each have a single exon. This analysis uncovered diversity in intron–exon structure across the UXS family while highlighting the conserved genomic architecture of *NnUXS* genes. BLAST analysis revealed that this gene family possesses the characteristic motif sequences of the UXS protein family: GXXGXXG and YXXXK (Figure 1E).

### 2.4. Analysis of Cis-Acting Elements in the Promoters of NnUXS Family Genes

**Figure 2:**
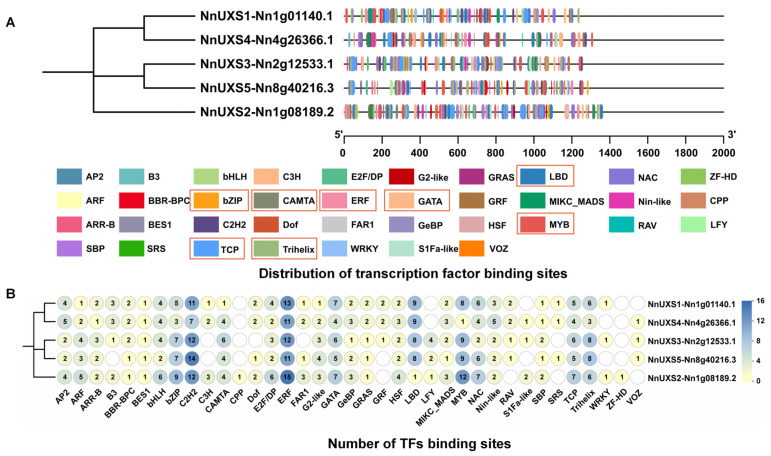
TFs binding site in *NnUXS* family genes. (**A**) Distribution of transcription factor binding sites, with distinct sites shown as colored boxes. Red box indicates transcription factors responsive to salt stress (**B**) Number of TFs binding sites.

Promoters are pivotal for transcriptional regulation, largely depends on cis-acting elements located upstream of genes [9,40]. Transcription factors (TFs) are critical regulators of plant gene expression, modulating key developmental processes by binding to specific DNA sequences within promoter regions. Accordingly, we analyzed TF binding sites in the promoters of *NnUXS* genes (Figure 2). The analysis revealed a diverse repertoire of enriched TF binding sites, including C2H2, bHLH, LBD, bZIP, CAMTA, ERF, MYB, TCP, Trihelix, and NAC, all of which are associated with growth and abiotic stress responses. Notably, the majority of these TFs are implicated in salt stress regulation, such as ERF (62 binding *cis*-elements), bZIP (31), CAMTA (19), GATA (29), MYB (39), TCP (27), and Trihelix (31) [41,42,43,44,45,46,47], suggesting that *NnUXS* family genes may play crucial roles in plant growth and salt stress tolerance.

Furthermore, 2000 bp upstream regions of *NnUXS* genes were examined to predict putative cis-acting elements (Figure S2). Two major classes of elements were identified: those related to growth and development-related elements and stress response. For growth and development (Figure S2A,E), four functional categories were detected, including light response, zein metabolism regulation, circadian control, and meristem expression. Their core motifs (Figure S2B) included G-box (54%), TCT-motif (12%), O2-site (9%), BOX I (9%), CATT (7%), ATCT (5%). Regarding stress response elements (Figures S3C), four types were identified, responding to anaerobic conditions, drought, wound, and low temperature. The core stress-related motifs (Figure S3D) comprised ARE (37%), MBS (18%), LTR (18%), GC-motif (18%), and WUN-motif (9%), with anaerobic response elements being the most abundant.

Together, these findings suggest that *NnUXS* genes are likely involved in mediating lotus growth and development, as well as in adaptive responses to various abiotic stresses, underscoring their potential role in modulating plant architecture and salt tolerance.

### 2.5. Gene Replication and Synteny Analysis of UXS Family Genes

**Figure 3:**
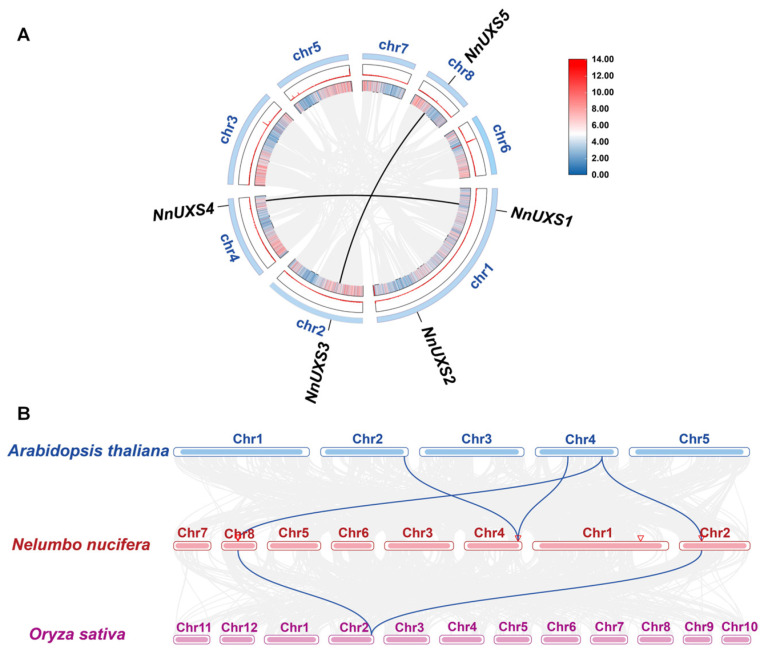
Gene duplication, and synteny of *NnUXS* genes. (**A**) Gene replication of *NnUXS* genes on eight chromosomes of *N. nucifera* genome. Gray lines represent the collinear blocks between the two genomes, and black lines represent the pairwise replication of the *NnUXS* genes. The orange rectangular boxes represent the 8 chromosomes. The middle and inner layer shows gene density distribution, indicated by the heatmap and peak height. (**B**) Synteny analysis of *UXS* genes between *N. nucifera*, *A. thaliana* and *O. sativa*, with blue lines highlighting syntenic regions.

We analyzed intraspecific gene duplications among the 5 *NnUXS* genes, identifying two pairs of segmental duplications in the *N. nucifera* genome: one localized between Chr2 (*NnUXS3*) and Chr8 (*NnUXS5*), and the other between Chr1 (*NnUXS1*) and Chr4 (*NnUXS4*) (Figure 3A). This reflects a degree of evolutionary conservation of the *NnUXS* family within the *N. nucifera* genome.

Comparative syntenic analyses were then performed between *N. nucifera* and two representative plant species: *A. thaliana* and *O. sativa*. These analyses uncovered 4 pairs of syntenic homologs between *N. nucifera* and *A. thaliana*, and 2 pairs between *N. nucifera* and *O. sativa*. Notably, lotus Chr2 and Chr8 exhibited synteny with both *A. thaliana* and *O. sativa*, while Chr4 showed synteny exclusively with *A. thaliana* (Figure 3B). These results suggest that *NnUXS* genes likely retain similar biological functions to their homologs and highlight that *N. nucifera* preserves more pronounced dicot characteristics—with a closer evolutionary relationship to *A. thaliana*. Furthermore, *NnUXS3* (on Chr2) *and NnUXS5* (on Chr8) may represent a more conserved and functionally critical member of the *NnUXS* family.

### 2.6. Structures Prediction and Molecular Docking of NnUXS Family Members

**Figure 4:**
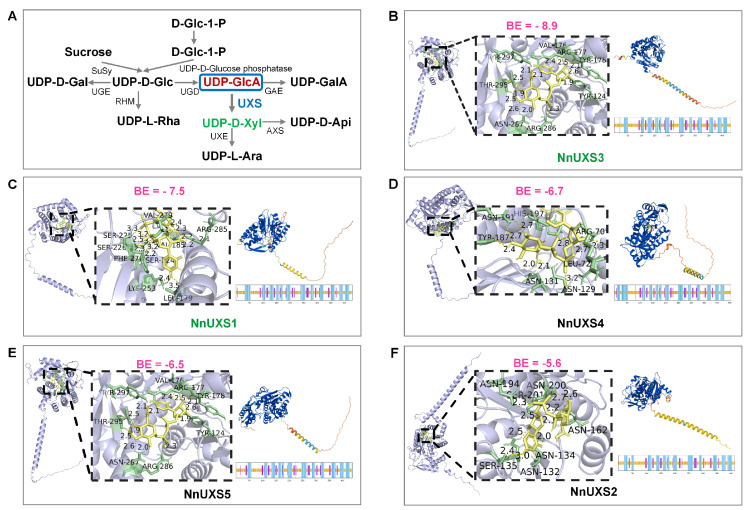
Structural prediction and molecular docking of NnUXS protein. (**A**) Schematic diagram of the biosynthetic pathway for UDP-sugar donors. (**B**–**F**) Molecular docking analyses of key functional domains of NnUXS with its optimal substrate UDP-GlcA; each subplot is divided into two panels: the left panel displays the full-length protein–ligand complex structure, and the right panel provides an enlarged view of the binding interface, with yellow dashed lines indicating hydrogen bonds and negative binding energy (BE) values (annotated in dashed boxes) quantifying binding affinity (larger absolute values correspond to stronger molecular interactions). The right section of the figure shows predicted secondary and 3D structures of the five NnUXS proteins: the top row displays 3D structural models, and the bottom row shows secondary structure diagrams, where blue, purple, and brown represent α-helices, extended strands, and random coils, respectively, in the secondary structure diagrams, and in the 3D structure visualizations, blue indicates ≥ 70% structural confidence, yellow represents 60–70% confidence, and red signifies < 50% confidence.

Grounded in the principle that protein structure dictates function, we first analyzed the secondary structure of five NnUXS proteins using the SOPMA tool to elucidate their functional potential (Figure 4 and Table S4). Results revealed that NnUXS proteins are predominantly composed of α-helices, extended strands, and random coils—with roughly equal proportions of these elements. Notably, α-helices and random coils were the most abundant, whereas extended strands were less prevalent, and β-sheets were completely absent (consistent with the definition of secondary structure elements: α-helices, β-sheets, β-turns, extended strands, and random coils). Next, we predicted the tertiary structure of NnUXS proteins via the Phyre2 threading algorithm (de novo modeling). The results showed substantial variability in their 3D architectures; notably, except for NnUXS1 (right panel of Figure 4F), the other four proteins (right panels of Figure 4B–E) exhibited relatively high structural similarity.

Furthermore, we performed molecular docking between the five NnUXS proteins (left panels of Figure 4B–F and Table S5) and their substrate UDP-GlcA (Figure 4A) by AlphaFold3. Binding energy (BE) analysis demonstrated that NnUXS3 (BE = −8.9 kcal/mol) and NnUXS1 (BE = −7.5 kcal/mol) had the highest affinity for UDP-GlcA, followed by NnUXS4 (BE = −6.7 kcal/mol). In contrast, NnUXS5 (BE = −6.5 kcal/mol) and NnUXS2 (BE = −5.6 kcal/mol) displayed markedly lower affinity. These findings suggest that NnUXS3 may play a relatively more critical functional role, likely serving as a key mediator in substrate binding and catalysis.

### 2.7. Identification of NnUXS3 as a Candidate Gene for Plant Architecture Dwarfing and Salt Tolerance in N. nucifera

**Figure 5:**
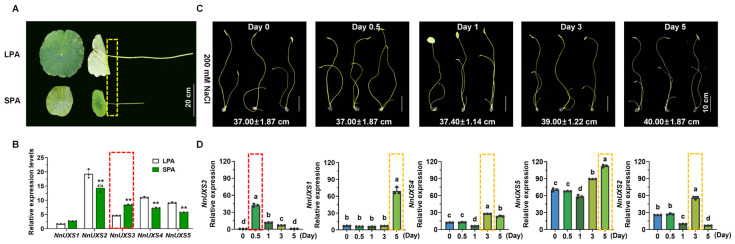
Expression patterns of *NnUXS* genes involved in plant architecture morphogenesis and aquatic salinity tolerance. (**A**) Phenotypic comparison of the LPA (large plant architecture) lotus ‘Geyue Lingyin’ and the SPA (small plant architecture) lotus ‘Xiao Lianzuo’. (**B**) qRT-PCR analysis of *NnUXS* genes expression in petioles of LPA and SPA varieties shown in (**A**), with *NnUBC* as the internal control (normalized to 100). Data represent means ± SD of three biological replicates. ** indicate significant differences between LPA and SPA varieties (*t*-test, *p* < 0.01, n = 3). (**C**) Phenotypic alterations of lotus seedlings under 200 mM NaCl treatment at 0, 0.5, 1, 3, and 5 days post-treatment. Means ± SD represent the length of petioles. (**D**) qRT-PCR analysis of *NnUXS* genes expression in lotus seedlings under NaCl treatment as shown in (**C**), with *NnUBC* as the internal control (normalized to 100). Data represent means ± SD of three biological replicates. Different letters indicate significant differences (*p* < 0.01) based on one-way ANOVA followed by Tukey’s test. To emphasize the peak expression time points, red or yellow dashed boxes are used.

Building on the above bioinformatic analyses to further link *NnUXS* family gene functions involved in modulating plant architecture and salt tolerance, we performed qRT-PCR to analyze *NnUXS* expression in the petioles of the lotus cultivars ‘Geyue Lingyin’ (large plant architecture, LPA) and ‘Xiao Lianzuo’ (small plant architecture, SPA), respectively (Figure 5A,B). Notably, only *NnUXS3* was significantly upregulated in SPA petioles, whereas *NnUXS2/4/5* were highly expressed in LPA varieties relative to SPA ones, and *NnUXS1* expression showed no significant difference across cultivars with distinct plant architectures.

Salinity, a major abiotic stressor, disrupts osmotic balance and nutrient uptake via excessive Na^+^, K^+^, and Cl^−^ accumulation, causing toxicity throughout plant growth (germination, seedling development, vegetative growth, flowering, and fruit set) [10]. This is particularly detrimental to lotus—a perennial aquatic species. Lotus seedlings exposed to 200 mM NaCl exhibited growth arrest and visible leaf wilting by day 5 (Figure 5C), confirming the substantial stress imposed by this treatment. qRT-PCR analysis of *NnUXS* genes expression in salt-stressed seedlings (0, 0.5, 1, 3, and 5 days post-treatment) revealed all *NnUXS* genes responded to salt stress (Figure 5D). notably, *NnUXS3* alone exhibited a peak in expression at 0.5 days—an early, rapid reaction distinct from other family members.

Collectively, these results identify *NnUXS3* as a candidate gene mediating both plant architecture dwarfing (via preferential expression in SPA varieties) and early salt stress response in *N. nucifera*.

### 2.8. Functional Validation of NnUXS3 in Plant Dwarfing and Lotus Salt Tolerance

**Figure 6:**
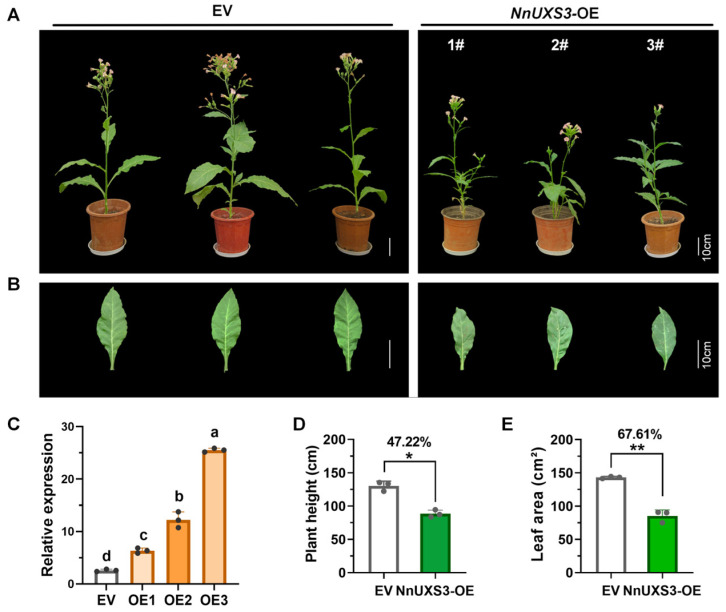
Phenotypic characterization of EV (Empty vector) and *NnUXS* -overexpressing (OE) tobacco lines. (**A**,**B**) Phenotypic comparison of whole-plant morphology and leaf traits between EV controls and *NnUXS*-OE transgenic lines at the mature stage. (**C**) Relative expression of *NnUXS3* gene in stems of EV and *NnUXS*-OE lines shown in (**A**), with data representing means ± SD from three biological replicates and different letters indicating significant differences (*p* < 0.01) based on one-way ANOVA followed by Tukey’s test. (**D**,**E**) Comparisons of plant height and leaf area between EV and *NnUXS*-OE lines at the mature stage, respectively, with data showing means ± SD from three biological replicates and * and ** denoting significant differences between EV and *NnUXS*-OE (*t*-test, *p* < 0.05 or *p* < 0.01, n = 3), along with percentage changes relative to EV. The number sign (#) denotes independent transgenic lines.

**Figure 7:**
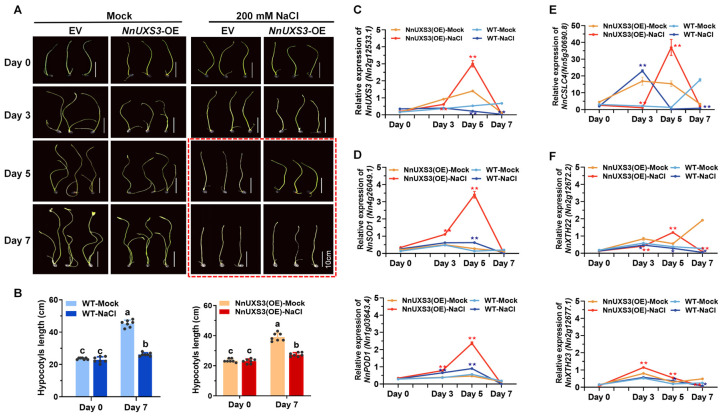
Effects of 200 mM NaCl treatment on transiently transformed lotus seedlings. (**A**) Phenotypic alterations of lotus seedlings under NaCl stress from 0 to 7 days post-treatment. (**B**) Comparison of analysis of petiole length between EV controls and *NnUXS*-OE lines under salt stress at 0 and 7 days. Data represent means ± SD of three biological replicates. Different letters indicate significant differences (*p* < 0.01) based on one-way ANOVA followed by Tukey’s test. (**C**–**F**) Temporal expression patterns of representative genes in transgenic plants under NaCl stress at 0, 3, 5, and 7 days. *NnUXS3* gene (**C**), *NnSOD1* and *NnPOD1* genes (**D**), cellulose synthase-like family gene *NnCSLC4* gene (**E**), and xyloglucan endotransglucosylase/hydrolase family genes *NnXTH*22 and *NnXTH*23 (**F**). Data represent means ± SD of three biological replicates. * and ** indicate significant differences between NaCl treatment and mock (*t*-test, *p* < 0.05 or *p* < 0.01, n = 3).

To experimentally validate the dual role of *NnUXS3* as a candidate for plant architecture dwarfing and salt tolerance in *N. nucifera*, we conducted heterologous overexpression in tobacco and transient overexpression in lotus seedlings (Figure 6 and Figure 7). An overexpression vector carrying *NnUXS3* was introduced into wild-type tobacco K326 (Figure 6 and Figure S1). qRT-PCR confirmed three independent transgenic lines (OE1, OE2, OE3) with significantly elevated *NnUXS3* transcript levels compared to the empty vector (EV) control (Figure 6C). Phenotypic analysis at the flowering stage revealed marked reductions in both plant height (Figure 6A) and leaf area (Figure 6B) in OE lines relative to EV. Quantitative measurements showed a 47.22% decrease in average plant height (Figure 6D) and a 67.61% reduction in leaf area (Figure 6E). These results directly demonstrate that *NnUXS3* overexpression drives plant dwarfing in tobacco.

To functionally validate *NnUXS3* in its native context, we employed an *Agrobacterium*-mediated transient overexpression system in lotus seedlings. Mirroring the dwarfing phenotype observed in transgenic tobacco, the transient overexpression of *NnUXS3* in lotus significantly inhibited the elongation of young petioles, an effect that was further accentuated under salt stress (Figure 7A,B). This result consolidates the conserved role of *NnUXS3* in regulating plant architecture. Furthermore, *NnUXS3* overexpression conferred a clear survival advantage under salinity. While empty vector (EV) control seedlings (WT-NaCl) exhibited pronounced wilting by day 5 post-treatment, *NnUXS3*-overexpressing lines (OE-NaCl) maintained turgor and remained visibly healthy until day 7 (Figure 7A,B), demonstrating enhanced salt tolerance.

The success of the transient transformation and the stress-induced expression of *NnUXS3* were confirmed by qRT-PCR. Consistent with the experimental design where salt treatment was initiated immediately after infiltration, transcript levels at Day 0 served as a pre-induction baseline and showed no significant difference between groups. Subsequently, *NnUXS3* expression was significantly upregulated in the overexpression lines (OE-NaCl and OE-Mock) compared to their respective wild-type controls (WT-NaCl and WT-Mock) at later time points under both mock and salt conditions (Figure 7C), confirming successful transgene induction. Stress-responsive gene analysis revealed that *NnSOD* and *NnPOD* (key antioxidant enzymes) peaked on day 5 of salt stress in both WT-NaCl and OE-NaCl, but OE-NaCl showed markedly higher expression (Figure 7D), suggesting *NnUXS3* boosts antioxidant capacity.

Further, we examined cell wall-related genes critical for salt tolerance: cellulose synthesis-like C4 (*NnCSLC4*), xyloglucan endotransglucosylase/hydrolases (*NnXTH22/23*), cellulose synthases (*NnCESA1/2-A*), and galacturonosyltransferases (*NnGAUT1/10*) [4,8,9,48]. In WT, these genes were largely unresponsive to salt stress. However, in OE-NaCl, *NnUXS3* (Figure 7C), *NnCSLC4* (Figure 7E), *NnXTH22* (Figure 7F), *NnCESA1* (Figure S3A), and *NnCESA2-A* (Figure S3B) were significantly induced, peaking on day 5 of salt stress (*NnXTH23* peaked on day 3, Figure 7F). *NnGAUT1/10* (pectin synthesis) remained unaffected (Figure S3C,D).

This indicates that *NnUXS3* overexpression remodels cell walls—specifically enhancing cellulose and hemicellulose synthesis—to strengthen structural integrity under salt stress. As UXS catalyzes UDP-glucuronic acid decarboxylation to UDP-xylose [18], a key step in hemicellulose production, our data suggest *NnUXS3* coordinates cell wall modification to confer salt tolerance, while its ectopic expression in tobacco directly reduces stature.

## 3. Discussion

### 3.1. Evolutionary Divergence and Functional Specialization of the Lotus UXS Gene Family in Aquatic Adaptation

The UDP-glucuronic acid decarboxylase (UXS) family, conserved across angiosperms for cell wall polysaccharide biosynthesis, has been extensively studied in model terrestrial plants like *Arabidopsis* and rice [18,49,50,51]. However, its evolutionary trajectory and functional adaptation in perennial aquatic plants—such as lotus (*N. nucifera*)—remain unexplored. This study reveals that the lotus UXS family (NnUXS1-NnUXS5) exhibits striking divergence from its dicot (*Arabidopsis*) and monocot (*rice*) orthologs (Figure 1A), reflecting adaptations to its unique ecological niche. We hypothesize that this divergence stems from lotus’s evolutionary history as an ancient aquatic dicot, subjected to distinct selective pressures (e.g., prolonged submergence, salinity fluctuations, and perennial growth). For instance, while *Arabidopsis UXS* genes are primarily involved in hemicellulose synthesis for aerial growth [23], lotus *UXS* may have evolved to prioritize cell wall modifications for both submerged vegetative growth and aerial reproductive development, explaining their functional differentiation. Structural prediction (Figure 4) further supports this hypothesis: NnUXS3 and NnUXS1, clustered phylogenetically, display the highest substrate affinity for UDP-GlcA (BE = −8.9 and −7.5 kcal/mol), critical for UDP-Xyl production, a key precursor for hemicellulose and pectin. This contrasts with NnUXS2, NnUXS2, and NnUXS4, which show minimal affinity, suggesting subfunctionalization: NnUXS3/1 may drive hemicellulose/pectin synthesis for stress response, while NnUXS2/4/5 regulate niche pathways (e.g., pectin remodeling for ion buffering in submerged tissues). This aligns with prior observations that UXS paralogs often evolve specialized roles to optimize resource allocation in response to environmental cues [6].

Functional divergence within the NnUXS family is further evidenced by their distinct expression profiles. Specifically, *NnUXS3* was markedly upregulated in the petioles of SPA lotus, whereas *NnUXS2/4/5* were predominantly expressed in LPA varieties (Figure 5). This variant-specific expression pattern, together with the rapid and early induction of *NnUXS3* under salt stress (peaking at 0.5 days, in contrast to the mid- or late-phase induction of other members) (Figure 5), positions *NnUXS3* as a key candidate coordinating growth (via cell wall modification) and stress response (via rapid activation under salinity).

This functional specialization is likely underpinned by the predicted subcellular localization of NnUXS proteins (Table 1), which aligns with the conserved division observed in model plants. Plant UXS enzymes are categorized into membrane-bound (typically Golgi-localized) and soluble cytosolic isoforms, channeling UDP-Xylose into distinct biosynthetic pathways. In Arabidopsis, Golgi-localized (UXS1, UXS2, UXS4) and cytosolic (UXS3, UXS5, UXS6) isoforms exist, with genetic evidence indicating that cytosolic UXS enzymes play a predominant role in xylan biosynthesis, as the *uxs3uxs5uxs6* triple mutant exhibits reduced Xyl content and irregular xylem morphology, while mutants of the Golgi-localized isoforms show no obvious phenotype [23]. In lotus, our predictions follow a similar compartmentalization pattern: NnUXS1 and NnUXS2, predicted to contain transmembrane helices and localize to the endoplasmic reticulum, represent the membrane-associated class, potentially involved in the synthesis of cell wall matrix polysaccharides within the secretory pathway. In contrast, NnUXS3 and NnUXS5 are predicted as cytosolic isoforms. Notably, NnUXS4 is predicted to localize to the nucleus, a distinct feature warranting further investigation. Given the critical role of cytosolic UXS isoforms in xylan biosynthesis established in Arabidopsis, the specific and early salt-stress induction of the cytosolic *NnUXS3* suggests it may drive a prioritized metabolic flux to produce UDP-Xylose for synthesizing hemicellulose components, such as xylan, which are crucial for rapid cell wall remodeling under stress. This specialized, stress-responsive role distinguishes it from the potentially more housekeeping or redundant functions of the membrane-associated NnUXS1/2. The co-existence of these compartmentalized isoforms within the lotus genome implies a sophisticated mechanism for partitioning nucleotide sugar precursors, fine-tuning cell wall biosynthesis to support both developmental programs and environmental adaptation.

### 3.2. NnUXS3 Mediated Growth-Defense Tradeoffs via Cell Wall Remodeling: Mechanistic Insights and Implications for Breeding

Our findings reveal species-specific functional evolution in the UXS family, moving beyond the canonical view of strictly conserved homologous gene functions. A core finding of this study is that heterologous overexpression of *NnUXS3* in tobacco results in reduced plant height and leaf area (Figure 6), a phenotypic outcome distinct from *OsUXS* mutants (e.g., rice *bc25*, which exhibits brittle stems and stunted growth [52]) and *AtUXS* mutants (which display impaired hemicellulose synthesis [23]). We attribute this discrepancy to phylogenetic and ecological divergence: despite conserved catalytic function, like *OsUXS3* and *AtUXS* [18], NnUXS3 catalyzes the critical step of UDP-GlcA decarboxylation to UDP-Xyl, essential for hemicellulose/pectin biosynthesis, its phenotypic impact diverges due to species-specific adaptations. Lotus, as a perennial aquatic plant, may have evolved UXS variants with distinct regulatory constraints: The high UDP-GlcA binding affinity of *NnUXS3* (Figure 4) may ensure robust UDP-Xyl supply, which in tobacco (a dicot with different growth habits) translates to accelerated cell wall stiffening that limits elongation, manifesting as reduced stature. In contrast, rice *OsUXS3* mutants show brittle stems because the upright growth of rice relies heavily on cellulose-rich cell walls, and reduced hemicellulose disrupts this structural balance [24]. Thus, while UXS catalyzes a conserved step in cell wall polysaccharide synthesis across species, its functional outcomes are shaped by phylogenetic lineage and ecological niche, highlighting species-specific evolution of UXS-mediated cell wall biology.

As sessile organisms, plants balance growth and stress tolerance via cell wall plasticity. Salt stress disrupts osmotic balance and ion homeostasis, necessitating rapid cell wall remodeling to maintain integrity. Our findings demonstrate that *NnUXS3* enhances salt tolerance by orchestrating a directed reprogramming of cell wall biosynthesis. In OE lines, key genes *NnCSLC4* (cellulose synthase-like [9]), *NnXTH22* (xyloglucan endotransglucosylase/hydrolase [4]), and *NnCESA1* (cellulose synthase [2]) were significantly upregulated, with expression peaking around day 5 of salt stress, coinciding with the pronounced phenotypic enhancement of tolerance (Figure 7 and Figure S3). This coordinated induction pattern underscores that cell wall remodeling is a temporally regulated process. The initial expression dynamics of individual genes, such as the transient variation observed for *NnCSLC4* at day 3, likely reflect the complex, phased nature of this network-level reprogramming rather than their ultimate functional contribution. Notably, *NnCSLC4*—a cellulose synthase-like gene previously identified as a salt-responsive candidate in lotus [9]—showed the strongest induction, suggesting a particularly important and synergistic role with NnUXS3.

This synergistic relationship likely operates at the metabolic level: NnUXS3 catalyzes the production of UDP-Xylose, a direct precursor for hemicellulose biosynthesis, while NnCSLC4 is predicted to utilize nucleotide sugars for synthesizing hemicellulose backbones [9]. Their coordinated action would therefore directly reinforce the cell wall matrix under salinity stress. This proposed mechanism is consistent with prior studies showing that OsUXS3 interacts with stress-responsive kinases to modulate tolerance [53] and that CSL genes are critical for salinity-induced adaptations [9]. The observed species-specific regulation may reflect distinct ecological adaptations—where aquatic lotus might prioritize such direct wall reinforcement, terrestrial plants could rely more on complex signaling cascades.

This growth-defense trade-off is further contextualized by the dual role of UDP-Xylose: as a key precursor for hemicellulose biosynthesis, it enhances mechanical strength, while its conversion into pectin components (e.g., via UDP-arabinose) contributes to ion homeostasis under stress [16,20]. The high substrate affinity of NnUXS3 likely ensures a robust supply of UDP-Xylose, enabling concurrent wall reinforcement and osmotic adjustment. Consequently, NnUXS3, together with its synergistic partner NnCSLC4, represents a high-priority target for molecular breeding, offering a strategic lever to coordinately improve plant architecture and salt tolerance in lotus and related crops.

Beyond its applied potential, this study expands the fundamental understanding of plant cell wall biology by demonstrating how UXS paralogs can retain conserved catalytic functions while evolving distinct regulatory integrations for environmental adaptation. Future work should prioritize a deeper mechanistic dissection, including fine-grained analysis of cell wall composition in *NnUXS3* transgenic lines and systematic elucidation of its regulatory network through interaction studies with other paralogs (e.g., NnUXS5) and downstream modifiers (e.g., XTHs, CSLs), to fully delineate this adaptive pathway.

### 3.3. Potential Complex Regulatory Networks of NnUXS3 Orchestrating Growth and Abiotic Stress Responses

The TF binding site analysis (Figure 2) uncovered enrichment of stress-responsive elements (ARE, MBS, LTR) and developmental motifs (G-box, TCT), implicating *NnUXS3* in transcriptional networks governed by TFs like WRKY, TCP, and ERF. This aligns with known TF roles that *MdWRKY9* suppresses brassinosteroid biosynthesis to induce dwarfing [54], while *AtTCP14/15* regulate internode elongation [55]. In rice, the *AP2/ERF* gene OsERF3 directly binds to WOX11 to regulate the elongation of crown roots [56]. In lotus, such TFs may integrate environmental cues (e.g., salinity) and developmental signals to fine-tune *NnUXS3* expression, balancing growth and stress adaptation.

Moreover, the early salt induction of *NnUXS3* and its interaction with stress-responsive TFs (e.g., ERF, MYB) position it as a hub in cross-talk between growth and defense pathways. This is consistent with studies showing that *OsSERF1* (rice) and *AtMYB41* (*Arabidopsis*) modulate stress tolerance via cell wall and ROS pathways [57,58]. Furthermore, the salt-tolerance-related transcription factor *GhERF13.12* has been identified in upland cotton (*Gossypium hirsutum*), and its overexpression in *Arabidopsis* has been shown to enhance salt tolerance in plants [59].

### 3.4. Advantages of Lotus Transient Transformation Technology and Its Application in Gene Function Verification

The absence of a robust, genotype-independent stable transformation system remains a major bottleneck for functional genomics in lotus, primarily due to its recalcitrant tissue culture and regeneration. Transient transformation technologies offer a powerful and rapid alternative to circumvent these limitations. In this study, the use of transient overexpression in lotus—exemplified by *NnUXS3* conferring salt tolerance and phenocopying the dwarfing complementation observed in stable transgenic tobacco—demonstrates the high reliability and efficiency of this approach for in planta gene function validation. This method operates directly on living tissues, bypassing the lengthy and often inefficient processes of dedifferentiation, selection, and regeneration required for stable transformation.

Transient transformation, particularly via injection-mediated delivery, represents a well-established technique in lotus [60,61]. This approach acts directly on living tissues, circumventing the lengthy processes of dedifferentiation and redifferentiation inherent to stable transformation, thereby offering markedly higher operational efficiency. It has been successfully employed to rapidly modulate phenotypic traits; for instance, transient overexpression of *NnMYB5* or *NnGST2* induces anthocyanin accumulation and visible petal reddening [60,61]. Building upon this foundation, our study demonstrates the extension of this approach to the functional analysis of abiotic stress tolerance. The broader utility of this platform lies in its versatility: transient overexpression not only enables gain-of-function studies, as exemplified here, but also paves the way for complementary loss-of-function analyses via transient silencing strategies (e.g., VIGS). Together, these approaches allow for comprehensive functional dissection of genes governing key traits—such as stress resilience, development, and adaptation—directly in the species of interest.

Thus, transient transformation effectively overcomes the fundamental constraint posed by the lack of efficient stable genetic systems in lotus and other recalcitrant species. It significantly accelerates the functional gene validation pipeline, from discovery to mechanistic insight, thereby directly facilitating and expediting molecular design breeding in these economically important but genetically challenging crops.

## 4. Material and Methods

### 4.1. Plant Materials and Sample Collection

The lotus materials utilized in this study were sourced from the National Lotus Germplasm Repository at Southwest Forestry University, Kunming. The tissue samples, which were petioles, were collected during the flowering season in the summer of 2025. The materials comprised two contrasting lotus cultivars: the LPA (large plant architecture) ‘Geyue Lingyin’ (petiole height > 50 cm, leaf diameter > 30 cm, petal diameter > 18 cm) and the SPA (small plant architecture) ‘Xiao Lianzuo’ (petiole height < 33 cm, leaf diameter < 24 cm, petal diameter < 12 cm) [4]. Upon collection, samples were immediately flash-frozen in liquid nitrogen and stored at −80 °C until further use.

### 4.2. Salinity Stress Treatment

To validate the response of the *NnUXS* genes to salt stress, lotus seedlings were exposed to 200 mM NaCl for 5 days [9]. For the salinity stress experiment, seedlings of the lotus variety ‘TKL 36’ were selected as the experimental material. Mature, plump seeds of uniform size were chosen, and the outer shell at the tail end was carefully trimmed. The seeds were then soaked in clean water, with the water changed 1–2 times daily. The soaking process was conducted under full light at 25–30 °C for approximately 15 days, until the first floating leaf was fully expanded. Healthy, uniformly growing seedlings were subsequently transferred to a 200 mM NaCl solution, with the water level maintained at about 5 cm, ensuring that the floating leaves remained fully above the water surface. Petioles (3–5 cm below the base of the first floating leaf) were collected at 0, 0.5, 1, 3, and 5 days after treatment, respectively. Untreated lotus seedlings were used as the control.

### 4.3. Identification and Property Analysis of NnUXS Family Genes

Data Acquisition: Download the Nelumbo genome, GFF, and protein files from the Nelumbo genome database (http://nelumbo.cngb.org/nelumbo/home (accessed on 1 August 2024)). Obtain the protein sequences of AtUXS (Table S1) from the TAIR database (https://www.arabidopsis.org/ (accessed on 1 August 2024)). Acquire the protein sequences of OsUXS (Table S2) from the Rice Genome Annotation Database (https://rice.plantbiology.msu.edu/index.shtml (accessed on 1 August 2024)). (2) Initial screening with bLAST: Utilize AtUXSs and OsUXS as seed sequences to perform a bidirectional BLAST search in TBtools 2.120, with a threshold set to 10-5, for the initial identification of NnUXS proteins. (3) The UXS protein features a Ser-Lys-Tyr catalytic triad [62,63]. We identified the conserved domains of the putative UXS genes using CDD (https://www.ncbi.nlm.nih.gov/Structure/bwrpsb/bwrpsb.cgi (accessed on 3 August 2024)). Redundant sequences were removed, and only those with intact domains were retained to obtain the UXS gene family members in lotus. The final sequences were nomenclatured according to their chromosomal locations (Table S3). (4) Protein property analysis: Analyze the amino acid composition, isoelectric point, and molecular weight of the NnUXS proteins using the online tool Protparam (https://web.expasy.org/protparam/ (accessed on 10 August 2024)). (5) Structure and localization prediction: Predict the transmembrane structures of the NnUXS proteins using TMHMM-2.0 (http://www.cbs.dtu.dk/services/TMHMM/ (accessed on 10 August 2024)). Determine the subcellular localization of these proteins using WoLF PSORT (https://wolfpsort.hgc.jp/ (accessed on 10 August 2024)) and CELLO (http://cello.life.nctu.edu.tw/ (accessed on 10 August 2024)).

### 4.4. Phylogenetic Analysis

We constructed a phylogenetic tree using the maximum likelihood (ML) method in MEGA 11 with 1000 bootstrap replicates, based on UXS protein sequences from *A. thaliana*, *O. sativa*, and *N. nucifera*. The tree was then visualized and refined using ChiPlot (https://www.chiplot.online/ (accessed on 15 August 2024)), using the software Adobe Illustrator 2020 (AI) to beautify phylogenetic trees.

### 4.5. Chromosome Localization, Motif Distribution and Gene Structure of NnUXS Family Genes

The Gene Location Visualization function in TBtools 2.120 was utilized to map the *NnUXS* family genes onto their respective chromosomes for visual representation. Next, conserved motifs within the UXS proteins were examined using the MEME Suite (https://meme-suite.org/meme/ (accessed on 15 August 2024)), allowing for the identification of up to 15 distinct motifs. Finally, the intron–exon structures of 5 *NnUXS* genes were analyzed using TBtools 2.120, leveraging genomic GFF data. Perform amino acid sequence alignment using DNAMAN software (DNAMAN Version 9).

### 4.6. Transcription Factor Binding Sites Analysis and Cis-Acting Elements Prediction of NnUXSs Promoter

The Fasta Extract function in TBtools 2.120 was utilized to obtain a 2000 bp upstream nucleotide sequence fragment (promoter sequence) from the lotus genome file for the *NnUXS* genes. This fragment was then submitted to the Plant CARE website (http://bioinformatics.psb.ugent.be/webtools/plantcare/html/ (accessed on 18 August 2024)) and PLACE database [64] for the prediction and annotation of cis-acting elements in the *NnUXS* gene promoters, facilitating functional classification and statistical analysis. Data visualization was conducted using TBtools 2.120 and GraphPad Prism 5.0. Additionally, the 2000 bp upstream nucleotide sequence fragments of the *NnUXS* genes were submitted to the Plant Transcriptional Regulatory Map (https://plantregmap.gao-lab.org/index-chinese.php (accessed on 18 August 2024)) for further prediction and analysis. The prediction results underwent classification and statistical analysis, with data visualization accomplished using the Graphics and Heat Map functions in TBtools 2.120 (Figure 2).

### 4.7. Gene Duplication and Synteny Analyses

In TBtools 2.120, the One Step MCScanX tool was utilized for synteny analysis within individual species and interspecies synteny analysis among *N. nucifera*, *O. sativa* and *A. thaliana*. Subsequently, the “Advanced Circos” tool was used to visualize the synteny plots of the *NnUXS* family genes in each species, which clearly displays the chromosomal locations of genes, the linkage relationships among them, and the collinear blocks formed by evolutionary events. The “Dual Synteny Plot of MCscanX” tool was employed to visualize the interspecies synteny plots.

### 4.8. Secondary, 3D Structure and Molecular Docking Analysis of NnUXS Family Genes

Protein structure of NnUXS proteins was predicted using the online tool Alphafold3 (https://alphafoldserver.com (accessed on 3 December 2025)) [65,66]. The predicted structures were validated with the PDBsum Generate tool (https://www.ebi.ac.uk/thornton-srv/databases/pdbsum (accessed 3 December 2025)), which is based on the PROCHECK program (https://www.ebi.ac.uk/thornton-srv/databases/pdbsum (accessed on 3 December 2025)). The structure file of the molecular docking substrate UDP-GlcA (CID: 16220076) was obtained from the PubChem database (https://pubchem.ncbi.nlm.nih.gov/ (accessed on 3 December 2025)). The SDF file was converted to PDB format using Open Babel GUI. AutoDock Vina v1.2.5 was employed for global docking with a grid box size of 25 × 25 × 25 Å (Ångstrom). Receptor and ligand files were prepared in AutoDockTools-1.5.7 (https://autodock.scripps.edu/ (accessed on 4 December 2025)), and binding energies (kcal/mol) were calculated for the top-ranked conformation. Results were visualized in PyMOL v3.0.4 (https://pymol.org/ (accessed on 4 December 2025)).

### 4.9. Nucleic Acid Isolation and qRT-PCR Analysis

Total RNA was isolated using the Eastep™ Super Total RNA Extraction Kit (Promega, Madison, WI, USA). cDNA synthesis was performed with 1 μg RNA using HiScript II Q RT SuperMix (Vazyme, Nanjing, China). qRT-PCR was conducted with SYBR^®^ Green Realtime PCR Master Mix-Plus (Takara, Tokyo, Japan) under the following conditions: polymerase activation for 30 s at 95 °C, followed by 40 cycles of 15 s at 95 °C, 15 s at 60 °C and 25 s at 72 °C. The *NnActin* (XM_010267616.1) gene served as an internal control, and gene expression was normalized to this reference gene. All primers used in these assays are listed in Table S6, and each assay was carried out with three biological replicates.

### 4.10. Construction of Overexpression Vectors and Genetic Transformation

The full-length CDS of the target gene was downloaded from the NCBI database. Gene-specific primers for homologous recombination were designed using Primer 5 software (Table S6). The overexpression vector used was pGWB418. The coding region of the *NnUXS3* gene was amplified and recovered using cDNA from the petioles of ‘Space Lotus’ seedlings as the template. Simultaneously, the pGWB418 vector was digested with *AfeI* and *SacI*, followed by recovery and purification. The recovered amplification product and the digested vector were ligated using the ClonExpress^®^ II One Step Cloning Kit (Nanjing, China) via a one-step recombination method. The ligation product was then transformed into *Escherichia coli* DH5α. Single clones were selected and verified, and after confirmation by sequencing, the plasmid was extracted and transformed into *Agrobacterium tumefaciens* GV3101 for subsequent genetic transformation in tobacco. A verified positive bacterial culture was inoculated at a 1:100 ratio into LB liquid medium containing 100 mg/L spectinomycin and 50 mg/L rifampicin, and incubated at 28 °C with shaking at 200 rpm until the OD_600_ reached 0.6–0.8. The tobacco plants were transformed using the leaf disc method [67], and the overexpression lines were subsequently identified (Figure S1). The expression level of the *NnUXS3* gene in the stems of positive transgenic lines was detected by qRT-PCR.

### 4.11. Transient Gene Expression in N. nucifera Seedlings

An Agrobacterium tumefaciens-mediated transient expression system was adapted for *N. nucifera* seedlings. The recombinant pGWB418 plasmid harboring the *NnUXS3* coding sequence (or the empty vector control) was transformed into A. tumefaciens strain GV3101. A single positive colony was inoculated in LB medium supplemented with appropriate antibiotics (50 mg/L kanamycin and 50 mg/L rifampicin) and cultured overnight at 28 °C with shaking at 220 rpm. The primary culture was used to inoculate a secondary culture at a 1:100 dilution and grown until the OD_600_ reached 0.8–1.0. Bacterial cells were harvested by centrifugation at 4000× *g* for 10 min at room temperature. The pellet was gently resuspended in an infiltration buffer (10 mM MgCl_2_, 10 mM MES, pH 5.6, 150 μM acetosyringone) to a final OD_600_ of 0.8. The bacterial suspension was then incubated at room temperature for 2–3 h without shaking prior to infiltration.

For the transient overexpression assay, uniformly grown 4-week-old lotus seedlings were used. *Agrobacterium tumefaciens* strain GV3101 harboring either the *NnUXS3*-overexpression vector or the empty vector (control) was prepared as a bacterial suspension (OD_600_ = 0.8) in infiltration buffer (10 mM MES, 10 mM MgCl_2_, 150 μM acetosyringone, pH 5.6). Using a sterile 1 mL syringe (without a needle), approximately 2 mL of the bacterial suspension was injected into the stem at a site about 5 cm below the first fully expanded floating leaf. Gentle pressure was applied until a water-soaked area became visible. Seedlings infiltrated with the *NnUXS3* overexpression construct were designated as the OE group, while those infiltrated with the empty vector served as the wild-type (WT) controls.

Immediately after infiltration, all seedlings (both OE and WT groups) were subjected to salt or mock treatments. The experiment comprised four groups: (1) WT + 200 mM NaCl (WT-NaCl), (2) OE + 200 mM NaCl (OE-NaCl), (3) WT + water (WT-Mock), and (4) OE + water (OE-Mock). Each group contained at least seven biological replicates (individual seedlings), and the entire experiment was independently repeated three times. Following treatment, plants were maintained under standard growth conditions (16/8 h light/dark cycle, 25 °C). Petiole samples from the infiltration zone were collected at 0, 3, 5, and 7 days post-treatment (dpt), immediately frozen in liquid nitrogen, and stored at −80 °C for subsequent RNA extraction and biochemical analyses.

## 5. Conclusions

**Figure 8:**
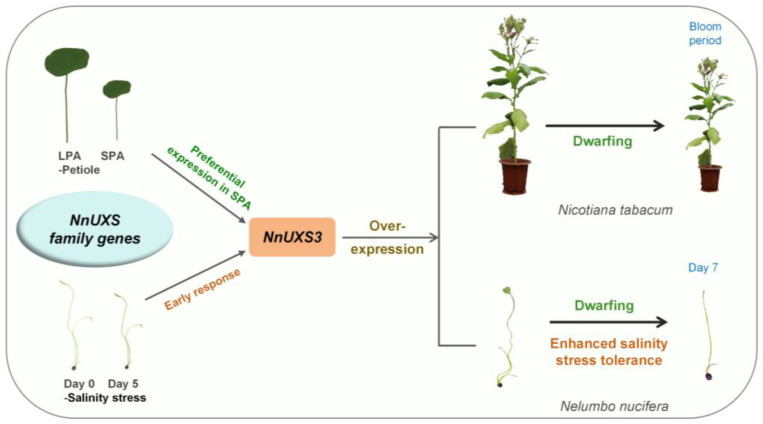
A proposed model of *NnUXS3* -mediated trade-off between plant dwarfing and salt tolerance. This model highlights the identification of *NnUXS3* as a key candidate gene from the *NnUXS* family, characterized by preferential expression in lotus SPA varieties and rapid induction under salt stress; Functional validation shows that heterologous overexpression in tobacco causes dwarfism, whereas transient overexpression in lotus enhances salt tolerance and reduces petiole length, supporting its role in balancing growth and stress adaptation.

This study presents a systematic characterization of the *NnUXS* gene family in *N. nucifera*, integrating genome-wide identification, evolutionary analysis, structural characterization, and functional validation. Evolutionary and synteny analyses with *A. thaliana* and *O. sativa* underscored the unique phylogenetic position of lotus. Comprehensive bioinformatic assessment of gene structures, conserved motifs, and promoter *cis*-elements implicated potential roles of NnUXS members in growth regulation and salt stress responses. From this family, *NnUXS3* emerged as a prime functional candidate, driven by its specific upregulation in SPA varieties, its rapid induction under salt stress, and its superior predicted binding affinity for UDP-GlcA. Functional assays confirmed its dual role: heterologous overexpression in tobacco significantly reduced plant height and leaf area, while transient overexpression in lotus enhanced salt tolerance and shorted petiole length. This improved resilience was mediated by the upregulation of key genes involved in cell wall remodeling (e.g., *NnCSLC4*, *NnXTH22*, and *NnCESA1*) and antioxidant enzymes (*NnSOD* and *NnPOD*). Collectively, our findings establish NnUXS3 as a critical mediator that orchestrates a trade-off between plant architecture and abiotic stress tolerance, highlighting its subcellular localization-driven role in tailoring cell wall remodeling for adaptation (Figure 8). This work not only identifies a valuable genetic target for breeding stress-resilient lotus but also provides broader insights into the UXS-mediated mechanisms balancing growth and environmental adaptation.

## Figures and Tables

**Table 1:** The information of the *UXS* family genes in *N. nucifera*.

Gene Name	Gene ID	LOC	AA (aa)	Mw (KDa)	PI	Instability Index	Aliphatic Index	GRAVY	TMHs	Subcellular Localization
*NnUXS1*	*Nn1g01140.1*	*LOC104602248*	414	46.005	10.11	50.72	82.2	−0.27	1	Endoplasmic reticulum
*NnUXS2*	*Nn1g08189.2*	*LOC104587515*	455	50.197	9.59	34.41	83.41	−0.251	1	Endoplasmic reticulum
*NnUXS3*	*Nn2g12533.1*	*LOC104612322*	433	47.831	9.62	33.24	79.28	−0.221	2	Cytoplasm
*NnUXS4*	*Nn4g26366.1*	*LOC104611461*	438	48.409	10	46.98	83.04	−0.226	1	Nucleus
*NnUXS5*	*Nn8g40216.3*	*LOC104605367*	431	47.671	9.73	36.65	80.12	−0.225	2	Cytoplasm

## Data Availability

The original contributions presented in this study are included in the article/Supplementary Materials. Further inquiries can be directed to the corresponding author.

## Supplementary Materials

The following supporting information can be downloaded at: https://www.mdpi.com/article/10.3390/plants15010116/s1, Figure S1: Identification of tobacco positive seedlings overexpressing NnUXS3. 1-5: Identification of positive transgenic tobacco plants overexpressing NnUXS3. ddH2O and EV represent the water control and empty vector control, respectively. Marker: BM2000+ DNA marker. Figure S2: Analysis of cis-acting elements in the NnUXS promoter regions. (A, B) Counts of cis-acting elements related to growth and development (A), and abiotic/biotic stress (B) identified in the NnUXS promoters. (C,D) Pie charts showing the proportional distribution of different cis-acting elements within each category: growth and development (C) and abiotic/biotic stress (D). (E) Total counts of all identified cis-acting elements. Figure S3: Temporal expression patterns of representative genes in transgenic plants under NaCl stress at 0, 3, 5, and 7 days. NnCESA1 (A) and NnCESA2-A gene (B), NnGAUT1 (C) and NnGAUT10 gene (D). Data represent means ± SD of three biological replicates. * and ** indicate significant differences between NaCl treatment and mock (*t*-test, *p* < 0.05 or *p* < 0.01, *n* = 3). Table S1: The amino acid sequences of AtUXS family. Table S2: The amino acid sequences of OsUXS family. Table S3: The amino acid sequences of NnUXS family. Table S4: Prediction of secondary structures for the NnUXS gene family. Table S5: Binding energy of molecular docking of key active components and core targets. Table S6: Primers used in this study.
