# Supplementary material for: Genome-Wide Analysis of Nelumbo nucifera UXS Family Genes: Mediating Dwarfing and Aquatic Salinity Tolerance
Source: Plants (Basel). 2026 Jan 1;15(1):116. doi: 10.3390/plants15010116 (PMC12787360; doi:10.3390/plants15010116)
Supplement: Supplementary file 1 [file plants-15-00116-s001.zip › R2 Wang L et al-Supply Figures (NnUXS) - 2025.12.19.pdf]

Genome-Wide Analysis of *Nelumbo nucifera UXS* Family Genes: Mediating Dwarfing and Aquatic Salinity Tolerance

Li Wang[1,†], Xingyan Zheng[1,†], Yajun Liu[2], Qian Mao[1], Yiwen Chen[1], Lin Zhao[3], Xiaomao Cheng[1], Longqing Chen[1,*] and Huizhen Hu[1,*]

Supplementary Figures

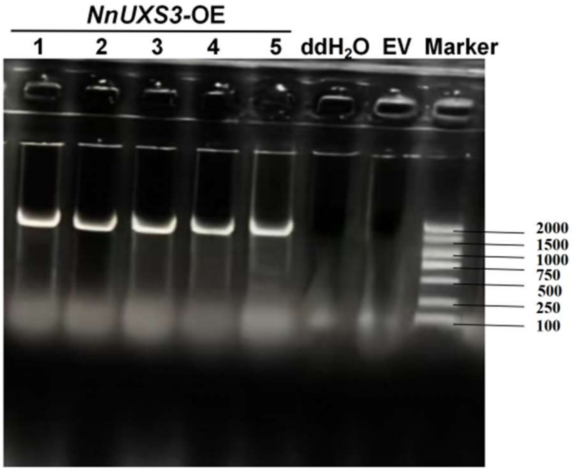

Figure S1 Identification of tobacco positive seedlings overexpressing *NnUXS3*. 1-5: Identification of positive transgenic tobacco plants overexpressing *NnUXS3*. ddH_2O and EV represent the water control and empty vector control, respectively. Marker: BM2000+ DNA marker.

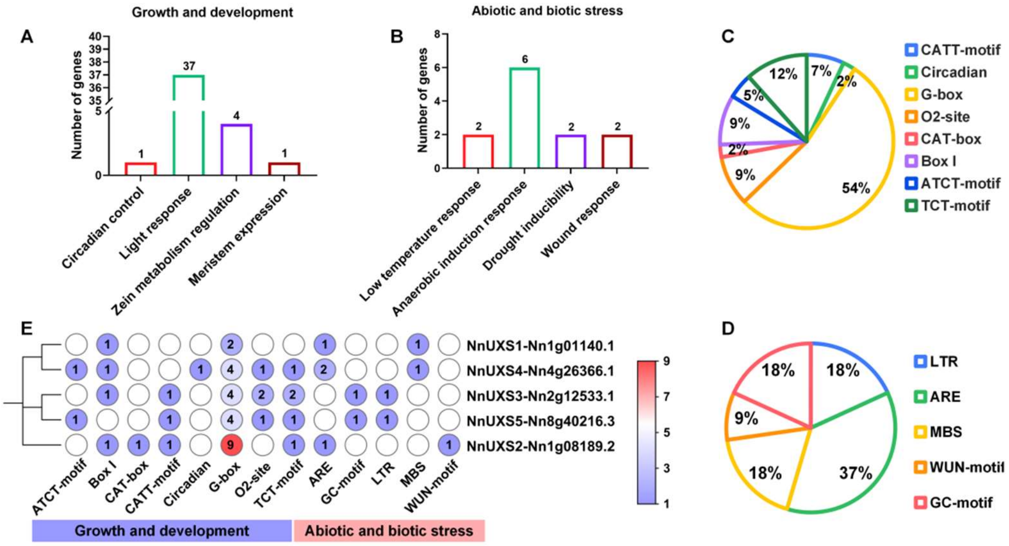

Figure S2. Analysis of cis-acting elements in the NnUXS promoter regions. (**A, B**) Counts of cis-acting elements related to growth and development **(A**), and abiotic/biotic stress (**B**) identified in the NnUXS promoters. (**C, D**) Pie charts showing the proportional distribution of different cis-acting elements within each category: growth and development (C) and abiotic/biotic stress (D). (**E**) Total counts of all identified cis-acting elements.

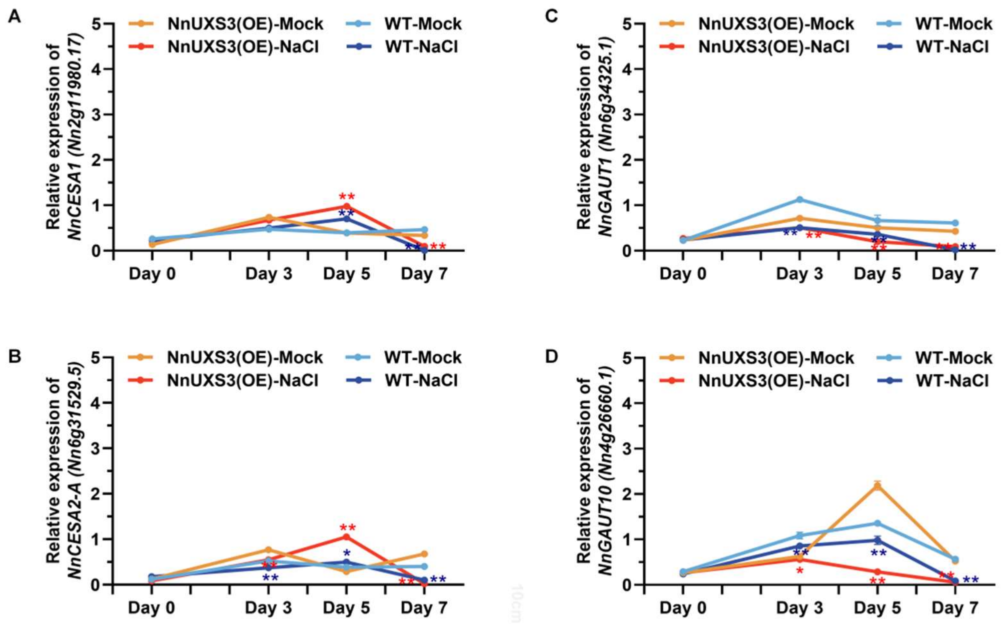

Figure S3. Temporal expression patterns of representative genes in transgenic plants under NaCl stress at 0, 3, 5, and 7 days. *NnCESA1* (A) and *NnCESA2-A* gene (B), *NnGAUT1* (C) and *NnGAUT10* gene (D). Data represent means ± SD of three biological replicates. * and ** indicate significant differences between NaCl treatment and mock (t-test, $P < 0.05$ or $P < 0.01$, n = 3).
